# Assessment of Attitudes, Main Concerns and Sources of Knowledge Regarding COVID-19 Vaccination in Poland in the Unvaccinated Individuals—A Nationwide Survey

**DOI:** 10.3390/vaccines10030381

**Published:** 2022-03-02

**Authors:** Mateusz Babicki, Wojciech Malchrzak, Agnieszka Mastalerz-Migas

**Affiliations:** Department of Family Medicine, Wroclaw Medical University, 51-141 Wroclaw, Poland; wojciech.malchrzak@gmail.com (W.M.); agnieszka.mastalerz-migas@umed.wroc.pl (A.M.-M.)

**Keywords:** COVID-19 vaccination, attitudes towards vaccination, COVID-19

## Abstract

Vaccination is the most effective tool to combat the COVID-19 pandemic. However, it is ineffective without appropriate public acceptance. In Poland, 53% of the country’s population is vaccinated, which puts us in the last position among the EU countries. Therefore, this study aims to assess the main concerns regarding vaccination in the unvaccinated population of Poland. The study was based on an original questionnaire that was distributed online. There were three phases of the study: Phase 1—before the preventive vaccination plan, Phase 2—2 months after implementation of the programme, Phase 3—after 4 months when the immunisation rate in Poland was 42%. A total of 4459 individuals participated in the study. As many as 1943 participants were excluded from the analysis due to lack of consent (30 subjects) or COVID-19 vaccination (1913 subjects). Out of the remaining 2516 unvaccinated individuals, 463 were participants in the first phase of the study, 1137 in the second phase of the study, and 916 in the third phase. As the preventive vaccination plan in Poland continued, concerns about vaccine adverse events, safety and efficacy were raised. The only lower concern was that about the vaccine transportation rules. Moreover, as the vaccination programme continued, there was an increase in the percentage of individuals declaring their full reluctance towards vaccination against COVID-19. Conclusions: The Internet is the main source of knowledge about the COVID-19 vaccination, so it should be focused on during vaccination campaigns. The public is primarily concerned about adverse events of vaccines and the lack of appropriate tests of the products used. Therefore, it is advisable to popularise the current state of knowledge and promote reliable information concerning the COVID-19 vaccination.

## 1. Introduction

Currently, the SARS-CoV-2 vaccination is the most effective way to prevent hospitalisations and deaths from COVID-19, a disease that has contributed to the deaths of nearly five million people out of more than 240 million detected infections [1]. Despite the efforts of scientists and health professionals from all over the world, many people are reluctant to get vaccinated, and some of them even openly criticise the COVID-19 vaccination by proclaiming opinions that are frequently inconsistent with scientific knowledge [2,3]. Those individuals often tend to base their views on diverse sources of information that are not always reliable.

The previous reports show that the main sources of knowledge concerning the COVID-19 vaccination include health professionals, the Internet and family members [4]. While relying on recommendations of health professionals is most frequently associated with greater willingness to vaccinate, it is not always the case when it comes to other sources [4]. This is largely due to omnipresent infodemics, both online and in everyday life [5]. In recent years, this phenomenon has been particularly evident in social media, where anyone can generate content without any oversight and, depending on the message, they can influence in various ways the attitudes towards the COVID-19 vaccination in the audience [6]. Such information is frequently related to the assessment of the safety, efficacy and appropriateness of vaccines, which are major concerns about the COVID-19 vaccination [7]. Despite the emergence of many studies that indicate both the high efficacy and safety profile of COVID-19 vaccines, a high level of these concerns is still observed, not only in Poland but also worldwide [8,9]. It has been proved that this may be due to a lack of appropriate information transfer. On the one hand, epidemiological studies reveal that reliable knowledge about vaccine adverse events is associated with a greater willingness to get vaccinated [7]. On the other hand, a lack of adequate information contributes to inducing vaccine-related fear [10]. In the case of the COVID-19 vaccination, confidence in scientific research and vaccine efficacy is also relevant. Doubts about this matter translate into less willingness to vaccinate [11]. As proven earlier, even the most effective medical remedy is not sufficient without appropriate social acceptance [5].

An adequate percentage of vaccinated people in a community can help produce herd immunity and reduce transmission of the virus in the community, and thus slow down the pandemic [12]. The exact values are not known and they are influenced by many factors including the virus variant and the efficacy of the vaccines administered. Currently, both in Poland and Europe, the Delta variant of COVID-19 is dominant and it is estimated that approximately 90% of the population need to be immunised to develop herd immunity [13]. Moreover, the emergence of another and, according to preliminary data, a highly infectious variant of COVID-19–Omicron may further increase the vaccination coverage level [14], which seems to be impossible in the current reality of Poland, where less than 56% of the population has been vaccinated [15]. However, individuals who have acquired immunity through natural contact with the virus should also be taken into consideration [16]. Furthermore, obtaining appropriate levels of vaccinated individuals may over time involve the possibility of lifting even a significant number of restrictions that protect against the COVID-19 infection. A good example is Portugal, where more than 90% of adult citizens are fully vaccinated—the government decided to return very quickly to the pre-pandemic rules. This state of affairs persisted until the end of November 2021, when some restrictions were reintroduced due to the emergence of the Omicron variant [17,18]. Currently, the number of infections in Portugal is increasing, which proves that even such a significant percentage of the vaccinated population does not completely stop the epidemic. Unfortunately, the duration of protection from infection provided by vaccination is shorter than originally anticipated and the population gets infected with both Delta and Omicron variants of COVID-19. Despite the considerable number of infected individuals in Portugal, the number of hospitalisations and deaths remains low, which proves the vaccine efficacy. Moreover, a consequence of the low percentage of the vaccinated population is naturally a higher number of infections, including those requiring hospitalisation and ending in death, especially in the risk group [19,20].

Another adverse event of under-vaccination is the continued circulation of the virus in the population of susceptible individuals, which causes further mutations of the virus. Some of these mutations may involve elements of the virus against which the vaccine-stimulated immune response is directed. Such an immune escape mechanism may reduce the previous vaccine efficacy and, in extreme cases, result in a lack of post-vaccination immunity [21,22].

Therefore, COVID-19 vaccination is currently a key pandemic control strategy, so it is extremely relevant to continuously monitor public sentiment and rapid local responses to improve it. Therefore, this study aims to assess the concerns that make people reluctant to get vaccinated against COVID-19 and their change over time. This study also aims to both determine demographic characteristics of vaccine refusers and the sources of knowledge they use, as well as identify potential changes throughout the epidemic. The distinguishing aspect of our study is the fact that the reports so far both from Poland and many countries around the world focus on one period. As we know, social moods may change over time, and our knowledge about vaccination also changes, which significantly affects the level of social acceptance. Therefore, to maintain the implementation of the vaccination program, it is necessary to constantly monitor the social mood and react appropriately based on scientific reports. The following research hypotheses were established: (1) As population vaccination continues, reluctance to COVID-19 vaccination in Poland is still at a high level and is even increasing. (2) Individuals who are not willing to get vaccinated are primarily concerned about vaccine-adverse events and lack of vaccine efficacy. (3) Opponents of vaccination derive their knowledge from unverified sources, mainly online ones. (4) Younger people with lower levels of education and those living in rural areas are less willing to get vaccinated.

## 2. Materials and Methods

The methodology of the study was based on an original questionnaire created for this project. The distribution of the questionnaire was via Facebook within groups concerning both COVID-19 vaccination, prophylactic vaccination and in general fora, with a total of nearly 30 groups being published. Questionnaire was powered by free Google Forms tool. The target group included individuals living in Poland, aged 18 and more. There was no sample size calculation and participants were collected randomly, but were always voluntary. Participation in the survey was fully voluntary and anonymous and respondents had the opportunity to withdraw from the study at any stage without giving any reason. The survey was visible and available to each member of the group in which the information was distributed. It was a cross-sectional study consisting of three successive stages. The first phase of the study was conducted over the period from 14 to 26 December 2020, before the implementation of the vaccination programme in Poland. The second phase of the study was conducted 2 months after the population vaccination was initiated in Poland when it was possible to vaccinate health professionals, individuals aged 70+ and younger with chronic, immunocompromising comorbidities [23,24,25,26]. The next follow-up period was from 16 June to 21 June 2021, when the vaccination rate was 42.28%, including 30.0% of individuals with a completed vaccination schedule. However, there was a gradual decline in the interest in vaccination among the Polish people, especially visible reluctance to receive a second dose. During this period, the COVID-19 vaccines were allowed in every Pole aged 12 and older [27]. During whole study time vaccination certificates ware not mandatory in the common sense. Only some modest benefits were connected with green passes such as not counting towards the limit of people in public place, but certificates never conditioned entering such places in general.

Before participation in the study, each respondent was informed about the nature of the study, its objectives, and after becoming familiar with the information, the participant gave their informed consent to participate in the study.

The study was approved by the Bioethics Committee of the Wroclaw Medical University and it was conducted in accordance with the Declaration of Helsinki.

The original questionnaire consisted of both single-choice and multiple-choice questions. The initial section included an assessment of the sociodemographic status of the respondents, including age, sex, place of residence, level of education, marital status and being a health professional. Immunisation history (no vaccinations/mandatory vaccinations/mandatory and recommended vaccinations) and individual experience of COVID-19 infection were also assessed. The major concerns associated with receiving the COVID-19 vaccine were also assessed, including the concern about lack of vaccine efficacy, lack of appropriate tests of the products, the concern about transport/storage of products, the concern about vaccine adverse events and future complications. Concerning public sentiment, subsequent concerns were sequentially added to the survey at various phases of the study. Moreover, in the second phase of the study, the questionnaire was extended to include main sources of knowledge about COVID-19 vaccination, including TV, Internet, doctor, health professional (other than doctor), friends and information leaflets. A question was also added to address public attitudes towards mandatory COVID-19 vaccinations. The question assessing willingness to get vaccinated against COVID-19 was phrased “Do you plan to get vaccinated against COVID-19?” and possible answers were “I am already vaccinated/Yes, as soon as possible/Yes, in a few months (up to a year)/Yes, in a year or more/I cannot make a decision/No, but I might consider it in the future/No, never”. The analysis in this study included the questionnaires of those participants who declared that they were not vaccinated against COVID-19.

### Statistical Analysis

The statistical analysis was performed using Statistica 13.0 software, StatSoft (Hamburg, Germany, StatSoft). Variables were of a qualitative and quantitative nature. Initially, basic descriptive statistics methods were used. The Pearson’s chi-squared test was used for determining the relationships between qualitative variables. The Cramér’s V coefficient or phi coefficient were additionally assessed. The analysis of covariance (ANCOVA) was performed to exclude the influence of potential confounding factors such as age, sex, place of residence, level of education, marital status and past COVID-19 infection.

The significance level of *p* = 0.05 was assumed in all tests that evaluated the statistical significance of the differences between mean values.

## 3. Results

This section may be divided by subheadings. It should provide a concise and precise description of the experimental results, their interpretation, as well as the experimental conclusions that can be drawn.

### 3.1. Characteristics of the Study Group

A total of 4459 individuals participated in the study. For the purposes of this study, 1943 participants were excluded from the analysis due to lack of consent (30 subjects) or COVID-19 vaccination (1913 subjects). Out of the remaining 2516 unvaccinated individuals, 463 were participants in the first phase of the study, 1137 in the second phase of the study, and 916 in the third phase. Young people, women, and residents of large cities predominated each phase of the study. The proportion of respondents who got infected with COVID-19 increased from phase to phase. When asked about their willingness to vaccinate, respondents most frequently indicated extreme answers: 32.35% of them are willing to get vaccinated as soon as possible, while 31.08% never want to get vaccinated. As the study continued, the proportion of those who opposed to the COVID-19 vaccination increased, most likely because those willing to vaccinate had already vaccinated and thus they were excluded from the study.

A detailed profile of the study participants by study phase is shown in Table 1.

### 3.2. Concerns about COVID-19 Vaccination

The participants indicated varied concerns about COVID-19 vaccination. Initially, the large number of people who were willing to receive the vaccine as soon as possible indicated concerns about vaccine adverse events, lack of appropriate tests, demanding transport of the vaccine or appropriate vaccine efficacy; however, fewer respondents reported such feelings as time went on. The situation is different for those who were not willing to get vaccinated—the longer the vaccines were available, the more often they reported concerns about them. Moreover, according to them, COVID-19 vaccination is linked to a conspiracy and is a medical experiment, and the virus itself is not dangerous—such concerns were hardly ever raised by those willing to get vaccinated. Considering all study groups over time, there was an increase in the proportion of participants who reported concerns about vaccine adverse events and about appropriate tests and vaccine efficacy. Only vaccine transportation issues were a concern that decreased over time. Detailed data concerning reported concerns by willingness to vaccinate and study phase are shown in Table 2. Potential confounding factors such as sex, age, place of residence and level of education did not affect any of the concerns analysed. Excluding concerns about the lack of appropriate tests of COVID-19 vaccines, the fact of being in the medical profession significantly affected the frequency with which respondents indicated a particular concern. In some cases (concern about vaccine adverse events and lack of appropriate tests), such an influence was correlated with marital status or vaccination history. There is also a strong correlation when vaccine efficacy is assessed by people of different marital status, and when there is concern about a conspiracy in individuals with different vaccination histories. A detailed summary of the analysis of covariance for potential confounding factors is shown in Table S1 that provides Supplementary Material for this study.

### 3.3. The Presence of Chronic Conditions and Main Sources of Knowledge Regarding COVID-19 Vaccination Compared to Willingness to Vaccinate

The second and third phases of the study included questions about illnesses reported by study participants. The detailed data are included in Table 3. While there were no statistically significant differences in the second phase of the study, participants with chronic diseases in the third study phase were more reluctant to get vaccinated. It should be noted that the participants with diagnosed oncological disorders were willing to get vaccinated as soon as possible in the second phase of the study. Interestingly, in the third phase of the study, most of the unvaccinated oncology patients were not willing to get vaccinated against COVID-19. Similarly, patients with mental and oncological disorders were more likely to report their reluctance to get vaccinated in the third phase of the study.

The respondents used various sources to gain knowledge about COVID-19 vaccines—complete information is summarised in Table 4. The Internet was most frequently declared a source of knowledge regarding COVID-19 vaccination. As the study continued, there was a definite negative impact of the Internet on the creation of anti-vaccine attitudes; in the third phase of the study, as many as 40% of previously unvaccinated respondents indicated the Internet as their source of knowledge. A similar phenomenon was observed in participants who indicated television as their main source of knowledge. There were no significant differences between the study groups in terms of the use of information provided by a doctor, other health care professional or patient information leaflets. The third phase of the study was extended to include a question concerning the use of scientific sources; this source of knowledge was the most frequently declared by participants who were not willing to get vaccinated, either temporarily or in general.

### 3.4. Impact of Sociodemographic Factors and the Willingness to Be Vaccinated against COVID-19

The impact of sociodemographic factors on the decision to get vaccinated against COVID-19 is presented in detail in Table 5. With most of the variables, a general trend of increasing percentage of people unwilling to vaccinate over time was noticeable. At each study phase, adolescent males demonstrated the lowest willingness to vaccinate. As the national COVID-19 vaccination programme continues, reluctance to vaccinate among unvaccinated health care workers increases. Past COVID-19 infection did not significantly influence the decision to vaccinate at any phase of the study. However, this decision was very significantly affected by previous vaccination history—those who had previously had other vaccinations, especially not only the mandatory ones, were more willing to vaccinate against COVID-19. Those who declared no vaccination in the past were particularly reluctant to vaccinate against COVID-19. Such individuals represented up to 80% of respondents in the third phase of the study.

## 4. Discussion

The aim of this study was to assess the variability of concerns over time and in the course of implementation of the national COVID-19 vaccination programme among respondents in Poland. As the population-based vaccination programme continued, an increase in the proportion of unvaccinated individuals in Poland reporting concerns about the efficacy and safety of vaccination versus vaccine adverse events was observed. The only concern that decreased over time was that of the transport of vaccines.

Furthermore, it was demonstrated that a considerable proportion of the population is still unable to make a decision or is planning to postpone vaccination. Additionally, a persistent reluctance to vaccinate among nearly 1/5 of the respondents is evident. The presence of chronic diseases as well as the sources of knowledge except for the Internet and television are not significant in the formation of attitudes towards vaccination, although in other studies the reliance on knowledge from health professionals influenced the willingness to vaccinate [4]. All these factors significantly affect the very slow increase in the percentage of the vaccinated in Poland [15].

Among those who never want to get a vaccine are mainly ‘anti-vaxxers’, that is, people who challenge the scientific value of vaccination. They undermine the role of vaccination in health care and exaggerate the risks of vaccine adverse events. Moreover, they accuse vaccination of adverse events that have not been scientifically linked to the vaccine [28]. At the same time, those are people who often deny the existence of a pandemic, stating that SARS-CoV-2 does not exist or is not as dangerous as it really is. This was also confirmed in the study we conducted, and among those who consider the pandemic as a conspiracy, more than 80% do not plan to ever be vaccinated, as do 65% of those not concerned about SARS-CoV-2. This attitude translates into a lack of adherence to recommendations, including a full unwillingness to vaccinate—all that makes such people pose a health risk to others [29,30]. They are most likely to represent a younger, female population, with lower levels of education, with no previous vaccination and no fear of COVID-19, which is consistent with the findings of other studies [31,32,33]. It was also confirmed in our observations where inhabitants of rural areas and those with lower education level most often declared total reluctance towards COVID-19 vaccination.

It is worth pointing out that out of those who are not vaccinated, 11% were health care workers. At each phase of the study, it was noticeable that the percentage of medical workers who do not want to be vaccinated was increasing; however, it should be noted that the data concerns only those not yet vaccinated, and the healthcare workers were vaccinated first, which means that the majority of those willing to vaccinate had already been vaccinated, and in the study group, an increasing percentage were vaccination opponents. In Poland, no official statistics on vaccination rates among medical workers are kept, while those in Europe, Hungary, Iceland and Ireland show that 100% of medical workers are vaccinated. In Denmark, Czech Republic and Malta, this percentage is also approaching 100%. At the other extreme stands Bulgaria with nearly 30% of healthcare workers vaccinated [17]. Currently, in Poland, vaccination against COVID-19 is not mandatory for anyone, including healthcare workers. The obligation of vaccination for health care workers has been introduced by such countries as Australia, Great Britain, France, Greece or New Zealand [34]. Moreover, in Poland, there were some initial announcements made concerning the introduction of mandatory vaccination among healthcare professionals, teachers and uniformed services [35].

In addition to anti-vaxxers, another group can be distinguished among those unwilling to vaccine, persons with doubts, who have concerns about vaccination that make them unable to make a final decision. Those individuals should become the target of public education efforts, with prior identification of factors deterring them from getting the vaccination [36]. While anti-vaxxers already have an established opinion about vaccination and it is very difficult to convince them to change their attitude, in the case of doubters, a reliable presentation of the facts and the benefit-risk balance may lead to a change in their mindset and thus a willingness to vaccinate against SARS-CoV-2.

At the time of the survey, there was a recognisable trend of unvaccinated individuals’ attitudes shifting towards indecision or complete aversion towards vaccination. This is probably due to the fact that those who were willing to get vaccinated have already got their vaccines, while the unconvinced individuals have been influenced by the anti-vaccine circles. Thus, those originally determined to vaccinate began to doubt, and those initially unconvinced became completely reluctant to vaccinate, among other things by amplifying the smoldering fears they had.

Of the concerns reported, some were related to the potential lack of vaccine efficacy—people who do not know what protection the vaccination offers are unwilling to undertake the risk if they believe they will not receive adequate protection [37]. Moreover, as vaccination of the population continues, the proportion of such people increases. This may be due to the fact that initially in Poland there was a governmental narrative that vaccination largely protects not only against the severe course of the disease but also against the disease itself. Currently, all EU-approved vaccines reduce the risk of severe course and death [20,38,39,40]. However, vaccinated people still develop the disease and some of them die despite vaccination [41]. These are mainly elderly people, burdened with chronic diseases, who initially constitute a group at high risk of COVID-19 incidence. This information is unacceptable for a part of the society and constitutes a basis for questioning the vaccine efficacy. This is corroborated by the replies of respondents who are primarily concerned that vaccines are not adequately tested and therefore do not provide the protection claimed by manufacturers. In addition, among the unvaccinated, there is also an increasing proportion of people directly pointing to a lack of vaccine efficacy as the main concern of COVID-19 vaccination. Inadequate and selective analysis of epidemiological data may amplify such a concern and thus perpetuate a negative attitude towards vaccination despite the fact that knowledge about it is quite substantial and the products themselves have been thoroughly tested [42].

Another argument encountered from people opting against vaccination is the fact of being convalescent, as these people assume that they will not contract COVID-19 again. As studies demonstrate that having recovered from COVID-19 infection does not exclude recurrence of the disease [43]. Moreover, the immunity thus acquired disappears faster than that induced by vaccination [44,45]. Considering also that vaccination is definitely safer than SARS-CoV-2 infection, it is important to educate the population also in this regard, so that the convalescent patients also get vaccinated.

An additional advantage of vaccination may be the reduction of virus transmissibility, although this has not been conclusively demonstrated in studies. As mentioned earlier, this is not the primary purpose of vaccination, but by making vaccinated individuals less likely to transmit the virus to others, an outbreak can be more easily controlled, and those who cannot get a vaccine for medical reasons can be protected [46,47,48].

It can also be observed that patients’ concerns have changed over time as new vaccine-induced complications were reported to the public, such as thromboembolic incidents in the case of vector vaccines or myocarditis incidents in the case of mRNA vaccines [42]. Despite the relative rarity of these vaccine-adverse events and the unwavering benefit-to-risk relation, people were more afraid of the potential occurrence of adverse sequelae of vaccination, including long-term complications, which, due to the short time since the introduction of vaccination, could not yet be clearly ruled out [49,50,51,52].

The only concern that was reported less frequently over time was that related to vaccine transport. The mRNA-based vaccines initially required transport and storage at low temperatures: −70 °C for Comirnaty and −20 °C for Spikevax. Such requirements were related to limited knowledge of mRNA stability at higher temperatures [53,54,55]. Over time, manufacturers declared that vaccines do not lose their properties as quickly at higher temperatures, and therefore the requirements for their transport and storage were successively relaxed [56]. This information was broadcast by the media which resulted in people being less concerned about potential problems with vaccine transport.

The Internet can be a very useful source of knowledge, but because of the abundance of information it contains, it can also be a dangerous source for someone who does not know how to verify the content they consult. Recently, there has been a noticeable increase in the activity of anti-vaccine movements, mainly on the Internet [57]. Social media groups that assemble people with common viewpoints become a sort of information bubble, which gives the members of such groups the impression of the universality of their opinions and limits their contact with people with different views [58].

Most surveys tested attitudes towards vaccination only once, without reassessment after a period of time, hence the limited ability to directly compare survey results. Williams et al. found a higher proportion of people willing to be vaccinated over time, at the same time as no significant difference in reported disincentives to vaccination [59]. In early 2021, a survey was conducted four times spaced one month apart in Poland, which showed no significant change in the proportion of people who do not want to get a vaccine. At the same time, the most frequently repeated argument against vaccination was the concern about vaccine adverse events [60]. In contrast, in the study by Aurilio et al. where Italian nurses did not observe that the concern about vaccine adverse events influenced the willingness to vaccinate. Instead, such an effect was observed in relation to the confidence in the vaccine efficacy [61].

Results from other studies on key factors are consistent with the findings we obtained. A history of influenza vaccination (which is a recommended vaccination in Poland) is a key factor that affects those willing to vaccinate against COVID-19, while concern about vaccine adverse events or lack of confidence in vaccine efficacy remain the primary factors associated with a lower willingness to vaccinate [62,63,64].

From a psychological perspective, the reason for the persistence of anti-vaccine attitudes may be the confirmation effect—participants in anti-vaccine communities present anecdotal evidence of the inefficacy or harmfulness of vaccines, while ignoring other explanations for the phenomenon in question, resulting in a misreading of the incidence of vaccination with the phenomenon as the cause thereof. This cognitive error is very common and in order not to commit it, scientific methods should be employed to assess the observed effects and the possible correlation between them [65,66].

Another justification is the phenomenon of reactance, that is, psychological resistance. People who are forced or forbidden to do something are more inclined to perform the opposite action, because it seems more attractive, regardless of whether it will be beneficial or harmful for that person [67,68]. Such is precisely the situation with regard to vaccinations, which, from an objective point of view, are beneficial for individuals. The greater the pressure to administer the vaccination, the greater the chance that the phenomenon of reactance will occur and the person will not opt for the vaccination, despite the fact that it will be disadvantageous for them; the phenomenon of freedom of choice, including apparent freedom, will be prioritised over health benefits.

The mass media are an enormous tool in providing reliable information to the public. Owing to a wide range of recipients, the media may create and reinforce pro-health attitudes, including pro-vaccination ones, by educating, verifying the information transmitted and combating the false information already propagated [69,70].

Another aspect to consider is making access to places and services dependent on the state of vaccination. Usually a certificate called “green pass” helps with this. In many countries a green pass is mandatory to access public places and it probably limits the spread of the epidemic [71]. In Poland, vaccinated people get a green pass, but it is rather unnecessary within the country. People use it when they go abroad or sometimes during some mass events. The government avoids introducing the general requirement of a green pass for unspecific reasons. In relation to the results of our study, a mandatory green pass could be considered as not directly connected to health benefits in favor of vaccination willingness.

The authors are aware of the limitations of this study. Undoubtedly, it is the methodology of data collection in the form of a questionnaire distributed via the Internet, which is subject to group selection bias. In order to compensate for this, the authors distributed the questionnaire to groups including not only extreme supporters and opponents of vaccination but also groups of general interest. Another limitation is the impossibility of estimating the number of people who received the questionnaire and the response rate. It should also be noted that the surveyed group is not representative for the Polish population due to the predominance of women, people of young age and those living in larger cities.

In conclusion, the analysis indicates a significant change in attitudes of previously unvaccinated people towards COVID-19 vaccination as the pandemic progresses, it identifies the most common concerns holding back the decision to vaccinate, and it highlights the most potentially effective tools for educating the public about vaccination. By understanding these factors, measures can be taken to increase the number of people willing to get a vaccine not only in Poland. The study’s results can be implemented by educational campaigns provided in any country. This is essential to achieve herd immunity, especially when new variants of the virus emerge—otherwise a lack of immunity will stimulate mutations creating new variants, which could sustain a death spiral locking humanity within a pandemic for many years.

## 5. Conclusions

Over time, a growing aversion to vaccination among the respondents was noticeable. This is a clear failure of vaccination education, promotion and popularisation in the country. The population is mostly concerned about the vaccine adverse events and the lack of appropriate tests. The main source of knowledge on vaccinations is the Internet, so it is the main resource to be focused on while launching campaigns encouraging people to get vaccinated. The public is primarily concerned about the vaccine adverse events and the lack of appropriate tests of the products used, so it is advisable to popularise the current state of knowledge and promote reliable information concerning the COVID-19 vaccination. At the same time, it should be emphasized that this survey is not fully comprehensive due to the fact that the surveyed group is not representative of the general population. Further research in this area is necessary.

## Figures and Tables

**Table 1:** Characteristics of the study group.

Variable		Phase 1 (*n* = 463) (%)	Phase 2 (*n* = 1137) (%)	Phase 3 (*n* = 916) (%)	Total (*n* = 2516) (%)
Sex	Male	114 (24.6)	222 (19.5)	360 (39.3)	696 (27.7)
	Female	349 (75.4)	915 (80.5)	556 (60.7)	1820 (72.34)
Age	18–29	183 (39.5)	626 (55.1)	279 (30.5)	1088 (43.2)
	30–39	131 (28.3)	315 (27.7)	333 (36.4)	779 (31.0)
	40–59	127 (27.4)	153 (13.5)	269 (29.4)	549 (21.8)
	>59	22 (4.8)	43 (3.8)	35 (3.8)	100 (4.0)
Place of residence	City > 250 k population	177 (38.2)	471 (41.4)	387 (42.2)	1035 (41.1)
	City/town of 50 k–250 k population	103 (22.2)	215 (18.8)	196 (21.4)	513 (20.4)
	Town of up to 50 k population	87 (18.8)	182 (16.0)	152 (16.6)	421 (16.7)
	Rural area	96 (20.7)	270 (23.7)	181 (19.8)	547 (21.7)
Level of education	Higher (university degree)	303 (65.4)	687 (60.4)	648 (70.7)	1638 (65.1)
	Secondary	136 (29.4)	397 (34.9)	226 (24.7)	759 (30.2)
	Vocational	18 (3.9)	35 (3.1)	23 (2.5)	76 (3.0)
	Lower secondary	5 (1.1)	8 (0.7)	10 (1.1)	23 (0.9)
	Primary	1 (0.2)	10 (0.9)	9 (1.0)	20 (0.8)
Marital status	Married	212 (45.8)	461 (40.5)	537 (58.6)	1210 (48.1)
	Domestic partnership/informal relationship	147 (31.7)	388 (34.1)	206 (22.5)	741 (29.5)
	Single	104 (22.5)	288 (25.3)	173 (18.9)	565 (22.5)
Health professional	Yes	108 (23.3)	82 (7.2)	87 (9.5)	277 (11.0)
	No	355 (76.7)	1055 (92.8)	829 (90.5)	2239 (89.0)
Diagnosed with COVID-19	No	389 (84.0)	938 (82.5)	685 (74.8)	2012 (80.0)
	Yes, in the past	69 (14.9)	179 (15.7)	230 (25.1)	478 (19.0)
	Yes, currently	5 (1.1)	20 (1.8)	1 (0.1)	26 (1.0)
Previous vaccinations	None	24 (5.2)	56 (4.9)	61 (6.7)	141 (5.6)
	Only mandatory	251 (54.2)	700 (61.6)	614 (67.0)	1565 (62.2)
	Mandatory and recommended	188 (40.6)	381 (33.5)	241 (26.3)	810 (32.2)
Chronic diseases	Yes	—	381 (33.5)	292 (31.9)	673 (32.8)
	No	—	755 (66.4)	624 (68.1)	1379 (67.2)
Opinion on mandatory vaccinations against COVID-19	For	—	372 (32.7)	88 (9.6)	460 (22.4)
	Against	—	580 (51.0)	761 (56.8)	1341 (65.4)
	No opinion	—	184 (16.2)	67 (7.3)	251 (12.2)
Willingness to vaccinate against COVID-19	Yes, as soon as possible	171 (36.9)	509 (44.8)	134 (14.6)	814 (32.4)
	Yes, in a few months	52 (11.2)	66 (5.8)	48 (5.2)	166 (6.6)
	Yes, in a year or more	12 (2.6)	22 (1.9)	9 (1.0)	43 (1.7)
	No, but I might consider it in the future	81 (17.5)	209 (18.4)	269 (29.4)	559 (22.2)
	No, never	113 (24.4)	254 (22.3)	415 (45.3)	782 (31.1)
	I am not able to make a decision	34 (7.3)	77 (6.8)	41 (4.5)	152 (6.0)

**Table 2:** Concerns about COVID-19 vaccination.

Concerns about COVID-19 Vaccination	Study Phase	Willingness to Vaccinate (*N*(%))								*p*	Cramér’s V
		Percentage of Individuals with Concerns [*N*(%)]	*p*	Yes, as Soon as Possible	Yes, in a Few Months	Yes, in over a Year	No, I Will Consider It in the Future	No, Never	I Am Not Able to Make a Decision		
Vaccine adverse event	1	240 (51.8)	0.025	57 (23.8)	26 (10.8)	8 (3.3)	53 (22.1)	69 (28.8)	27 (11.3)	<0.001	0.311
	2	612 (53.8)		198 (32.4)	37 (6.1)	15 (2.5)	148 (24.2)	153 (25.0)	61 (10.0)	<0.001	0.291
	3	537 (58.6)		63 (11.7)	28 (5.2)	5 (0.9)	168 (31.3)	241 (44.9)	32 (6.0)	0.008	0.130
Vaccines have not been appropriately tested	1	235 (50.8)	<0.001	35 (14.9)	30 (12.8)	11 (4.7)	66 (28.1)	71 (30.2)	22 (9.4)	<0.001	0.491
	2	523 (46.0)		77 (14.7)	31 (5.9)	12 (2.3)	162 (31.0)	181 (34.6)	60 (11.5)	<0.001	0.575
	3	583 (63.6)		30 (5.2)	24 (4.1)	6 (1.0)	219 (37.6)	275 (47.2)	29 (5.0)	<0.001	0.393
Transport	1	122 (26.3)	<0.001	53 (43.4)	15 (12.3)	4 (3.3)	19 (15.6)	23 (18.9)	8 (6.6)	0.422	0.103
	2	175 (15.4)		73 (41.7)	13 (7.4)	5 (2.9)	29 (16.6)	36 (20.6)	19 (10.9)	0.154	0.084
	3	116 (12.7)		10 (8.6)	5 (4.3)	1 (0.9)	35 (30.2)	62 (53.5)	3 (2.6)	0.256	0.084
Vaccine efficacy	1	98 (21.2)	<0.001	26 (26.5)	7 (7.14)	1 (1.0)	21 (21.4)	38 (38.8)	5 (5.1)	0.002	0.203
	2	341 (30.0)		71 (20.8)	23 (6.7)	9 (2.6)	92 (27.0)	116 (34.0)	30 (8.8)	<0.001	0.320
	3	307 (33.5)		27 (8.8)	8 (2.6)	4 (1.3)	107 (34.9)	145 (47.2)	16 (5.2)	<0.001	0.158
The COVID-19 pandemic is a conspiracy	1	66 (14.3)	0.095	0 (0.0)	0 (0.0)	0 (0.0)	7 (10.6)	58 (87.9)	1 (1.5)	<0.001	0.609
	2	141 (12.4)		1 (0.7)	2 (1.4)	0 (0.0)	24 (17.0)	113 (80.1)	1 (0.7)	<0.001	0.537
	3	144 (15.7)		1 (0.7)	1 (0.7)	0	37 (25.7)	103 (71.5)	2 (1.4)	—	—
Other	1	13 (2.8)	<0.001	8 (61.5)	0 (0.0)	0 (0.0)	2 (15.4)	3 (23.1)	0 (0.0)	0.408	0.105
	2	76 (6.7)		12 (15.8)	1 (1.3)	2 (2.6)	19 (25.0)	40 (52.6)	2 (2.6)	<0.001	0.221
	3	90 (9.8)									
COVID-19 vaccination is a medical experiment	1	--	----	—	—	—	—	—	—	—	—
	2	--		—	—	—	—	—	—	—	—
	3	257 (28.1)		0 (0.0)	0 (0.0)	0 (0.0)	79 (30.7)	176 (68.5)	2 (0.8)	<0.001	0.374
SARS-CoV-2 is not dangerous	1	--	---	—	—	—	—	—	—	—	—
	2	--		—	—	—	—	—	—	—	—
	3	158 (17.2)		0 (0.0)	1 (0.6)	0 (0.0)	50 (31.7)	104 (65.8)	3 (1.9)	<0.001	0.253
Symptoms that may occur in the future	1	--	----	—	—	—	—	—	—	—	—
	2	--		—	—	—	—	—	—	—	—
	3	581 (63.4)		49 (8.4)	27 (4.7)	6 (1.0)	203 (35.0)	267 (46.0)	29 (5.0)	<0.001	0.257

**Table 3:** Chronic diseases in the context of willingness to be vaccinated against COVID-19.

Variable	Phase	Willingness to Vaccinate (*N*(%))						*p*	Cramér’s V
		Yes, as Soon as Possible	Yes, in a Few Months	Yes, in over a Year	No, I Will Consider It in the Future	No, Never	I Am Not Able to Make a Decision		
Chronic conditions	1	—	—	—	—	—	—	—	—
	2	192 (50.4)	19 (5.0)	8 (2.1)	60 (15.8)	77 (20.2)	25 (6.6)	0.141	0.085
	3	63 (21.6)	24 (8.2)	4 (1.4)	78 (26.7)	107 (36.6)	16 (5.5)	<0.001	0.184
Cardiovascular diseases	1	—	—	—	—	—	—	—	—
	2	38 (51.4)	3 (4.1)	2 (2.7)	14 (18.9)	14 (18.9)	3 (4.1)	0.741	0.049
	3	11 (16.7)	3 (4.6)	0 (0.0)	21 (31.8)	24 (36.4)	7 (10.6)	0.139	0.095
Respiratory diseases	1	—	—	—	—	—	—	—	—
	2	34 (53.1)	2 (3.1)	1 (1.6)	11 (17.2)	11 (17.2)	5 (7.8)	0.710	0.051
	3	4 (8.5)	2 (4.3)	1 (2.1)	10 (21.3)	26 (55.3)	4 (8.5)	0.304	0.081
Neurological diseases	1	—	—	—	—	—	—	—	—
	2	10 (55.6)	0 (0.0)	0 (0.0)	2 (11.1)	5 (27.8)	1 (5.6)	0.745	0.049
	3	7 (38.9)	1 (5.6)	0 (0.0)	5 (27.8)	5 (27.8)	0 (0.0)	0.086	0.103
Oncological diseases	1	—	—	—	—	—	—	—	—
	2	9 (100.0)	0 (0.0)	0 (0.0)	0 (0.0)	0 (0.0)	0 (0.0)	0.047	0.099
	3	4 (36.4)	0 (0.0)	0 (0.0)	3 (27.3)	4 (36.4)	0 (0.0)	0.416	0.074
Mental disorders	1	—	—	—	—	—	—	—	—
	2	37 (46.3)	6 (7.5)	1 (1.3)	11 (13.8)	21 (26.3)	4 (5.0)	0.750	0.049
	3	16 (32.0)	6 (12.0)	0 (0.0)	10 (20.0)	12 (24.0)	6 (12.0)	<0.001	0.180
Skin diseases	1	—	—	—	—	—	—	—	—
	2	21 (44.7)	2 (4.3)	1 (2.1)	6 (12.8)	12 (25.5)	5 (10.6)	0.798	0.046
	3	8 (22.9)	1 (2.9)	0 (0.0)	7 (20.0)	16 (45.7)	3 (8.6)	0.430	0.073
Endocrine disorders	1	—	—	—	—	—	—	—	—
	2	82 (48.5)	11 (6.5)	5 (3.0)	32 (18.9)	29 (17.2)	10 (5.9)	0.476	0.063
	3	30 (22.9)	14 (10.7)	4 (3.1)	33 (25.2)	45 (34.4)	5 (3.8)	<0.001	0.174
Other diseases	1	—	—	—	—	—	—	—	—
	2	35 (43.8)	4 (5.0)	3 (3.8)	12 (15.0)	19 (23.8)	7 (8.8)	0.748	0.049
	3	15 (20.0)	4 (5.3)	0 (0.0)	20 (26.7)	29 (38.7)	7 (9.3)	0.172	0.092

**Table 4:** Sources of knowledge about vaccines and the willingness to vaccinate against COVID-19.

Variable	Phase	Percentage of Participants Using a Particular Source of Knowledge [*N*(%)]		Willingness to Vaccinate (*N*(%))						*p*	Cramér’s V
			*p*	Yes, as Soon as Possible	Yes, in a Few Months	Yes, in over a Year	No, I Will Consider It in the Future	No, Never	I Am Not Able to Make a Decision		
Internet	1	—	0.001	—	—	—	—	—	—	—	—
	2	915 (80.5)		423 (46.2)	56 (6.1)	20 (2.2)	163 (17.8)	190 (20.8)	63 (6.9)	0.052	0.098
	3	675 (73.7)		107 (15.9)	37 (5.5)	8 (1.2)	216 (32.0)	270 (40.0)	37 (5.5)	<0.001	0.186
Television	1	—	<0.001	—	—	—	—	—	—	—	—
	2	340 (29.9)		139 (40.9)	20 (5.9)	9 (2.7)	66 (19.4)	77 (22.7)	29 (8.5)	0.375	0.069
	3	186 (20.3)		20 (10.8)	18 (9.7)	3 (1.6)	62 (33.3)	65 (35.0)	18 (9.7)	<0.001	0.190
Doctor	1	—	0.063	—	—	—	—	—	—	—	—
	2	387 (34.0)		192 (49.6)	22 (5.7)	5 (1.3)	69 (17.8)	73 (18.9)	26 (6.7)	0.164	0.083
	3	358 (39.1)		60 (16.8)	20 (5.6)	4 (1.1)	90 (25.1)	167 (46.7)	17 (4.8)	0.305	0.081
Health professional	1	—	0.064	—	—	—	—	—	—	—	—
	2	421 (37.0)		188 (44.7)	25 (5.9)	6 (1.4)	92 (21.9)	85 (20.2)	25 (5.9)	0.195	0.081
	3	294 (32.1)		44 (15.0)	13 (4.4)	5 (1.7)	82 (27.9)	137 (46.6)	13 (4.4)	0.671	0.060
Information leaflets	1	—	0.932	—	—	—	—	—	—	—	—
	2	184 (16.2)		72 (39.1)	10 (5.4)	2 (1.1)	41 (22.3)	48 (26.1)	11 (6.0)	0.328	0.071
	3	154 (16.8)		23 (14.9)	5 (3.3)	2 (1.3)	43 (27.9)	70 (45.5)	11 (7.1)	0.456	0.071
Friends	1	—	0.013	—	—	—	—	—	—	—	—
	2	258 (22.7)		86 (33.3)	11 (4.3)	9 (3.5)	56 (21.7)	76 (29.5)	20 (7.8)	<0.001	0.149
	3	160 (17.5)		22 (13.8)	5 (3.1)	2 (1.3)	59 (36.9)	64 (40.0)	8 (5.0)	0.225	0.087
Other	1	—	<0.001	—	—	—	—	—	—	—	—
	2	241 (21.2)		440 (49.2)	56 (6.3)	18 (2.0)	163 (18.2)	157 (17.5)	61 (6.8)	<0.001	0.237
	3	132 (14.4)		122 (15.6)	45 (5.7)	7 (0.9)	236 (30.1)	337 (43.0)	37 (4.7)	0.013	0.126
Scientific sources	1	—		—	—	—	—	—	—	—	—
	2	—		—	—	—	—	—	—	—	—
	3	597 (65.2)		62 (10.4)	21 (3.5)	6 (1.0)	179 (30.0)	309 (51.8)	20 (3.4)	<0.001	0.237

**Table 5:** Impact of sociodemographic variables on attitudes towards COVID-19 vaccination.

Variable		Phase	Willingness to Vaccinate (*N*(%))						*p*	Cramér’s V
			Yes, as Soon as Possible	Yes, in a Few Months	Yes, in over a Year	No, I Will Consider It in the Future	No, Never	I Am Not Able to Make a Decision		
Sex	Male	1	32 (28.1)	11 (9.7)	4 (3.5)	27 (23.7)	36 (31.6)	4 (3.5)	0.019	0.171
		2	92 (41.4)	13 (5.68)	2 (0.9)	37 (16.7)	69 (31.1)	9 (4.1)	0.010	0.115
		3	21 (5.8)	7 (1.9)	5 (1.4)	131 (36.4)	191 (53.1)	5 (1.4)	<0.001	0.286
	Female	1	139 (39.8)	41 (11.8)	8 (2.3)	54 (15.5)	77 (22.1)	30 (8.6)	0.019	0.171
		2	417 (45.6)	53 (5.8)	20 (2.2)	172 (18.8)	185 (20.2)	68 (7.4)	0.010	0.115
		3	113 (20.3)	41 (7.4)	4 (0.7)	138 (24.8)	224 (40.3)	36 (6.5)	<0.001	0.286
Age	18–29	1	48 (26.2)	21 (11.5)	7 (3.8)	43 (23.5)	50 (27.3)	14 (7.7)	0.007	0.151
		2	251 (40.1)	36 (5.8)	13 (2.1)	138 (22.0)	139 (22.2)	49 (7.8)	0.050	0.086
		3	62 (22.2)	23 (8.2)	4 (1.4)	89 (31.9)	83 (29.8)	18 (6.5)	<0.001	0.168
	30–39	1	55 (42.0)	12 (9.2)	4 (3.1)	17 (13.0)	34 (26.0)	9 (6.9)	0.007	0.151
		2	154 (48.9)	23 (7.3)	6 (1.9)	44 (14.0)	70 (22.2)	18 (5.7)	0.050	0.086
		3	51 (15.3)	20 (6.0)	4 (1.2)	82 (24.6)	158 (47.5)	18 (5.4)	<0.001	0.168
	40–59	1	52 (41.0)	18 (14.2)	1 (0.8)	20 (15.8)	27 (21.3)	9 (7.1)	0.007	0.151
		2	79 (51.6)	6 (3.9)	3 (2.0)	23 (15.0)	35 (22.9)	10 (23.3)	0.050	0.086
		3	16 (6.0)	5 (1.9)	1 (0.4)	89 (33.1)	154 (57.3)	4 (1.5)	<0.001	0.168
	>59	1	16 (72.7)	1 (4.6)	0 (0.0)	1 (4.6)	2 (9.1)	2 (9.1)	0.007	0.151
		2	25 (58.1)	1 (2.3)	0 (0.0)	4 (9.3)	10 (23.3)	3 (7.0)	0.050	0.086
		3	5 (14.3)	0 (0.0)	0 (0.0)	9 (25.7)	20 (57.1)	1 (2.9)	<0.001	0.168
Place of residence	City > 250 k population	1	81 (45.8)	24 (13.6)	5 (2.8)	28 (15.8)	26 (14.7)	13 (7.3)	0.019	0.143
		2	254 (53.9)	23 (4.9)	10 (2.1)	80 (17.0)	83 (17.6)	21 (4.5)	0.001	0.106
		3	53 (13.7)	24 (6.2)	4 (1.0)	113 (29.2)	182 (47.0)	11 (2.8)	0.558	0.070
	City/town of 50 k–250 k population	1	81 (45.8)	24 (13.5)	5 (2.8)	28 (15.8)	26 (14.7)	13 (7.3)	0.019	0.143
		2	84 (39.3)	11 (5.1)	6 (2.8)	41 (19.2)	58 (27.1)	14 (6.5)	0.001	0.106
		3	32 (16.3)	4 (2.0)	2 (1.0)	56 (28.6)	92 (46.9)	10 (5.1)	0.558	0.070
	Town of up to 50 k population	1	27 (31.0)	10 (11.5)	1 (1.2)	18 (20.7)	24 (27.6)	7 (8.1)	0.019	0.143
		2	73 (40.1)	13 (7.1)	3 (1.7)	18 (9.9)	39 (21.4)	18 (9.9)	0.001	0.106
		3	20 (13.2)	9 (5.9)	1 (0.7)	42 (27.6)	69 (45.4)	11 (7.2)	0.558	0.070
	Rural area	1	26 (27.1)	5 (5.2)	4 (4.2)	17 (17.7)	36 (37.5)	8 (8.3)	0.019	0.143
		2	98 (36.3)	19 (7.0)	3 (1.1)	52 (19.3)	74 (27.4)	24 (8.9)	0.001	0.106
		3	29 (16.0)	11 (6.1)	2 (1.1)	58 (32.0)	72 (39.8)	9 (5.0)	0.558	0.070
Level of education	Higher (university degree)	1	137 (45.2)	36 (11.9)	9 (3.0)	50 (16.5)	53 (17.5)	18 (5.9)	<0.001	0.187
		2	319 (46.4)	46 (6.7)	14 (2.0)	132 (19.2)	130 (18.9)	46 (6.7)	0.005	0.093
		3	89 (13.7)	31 (4.8)	8 (1.2)	197 (30.4)	292 (45.1)	31 (4.8)	0.038	0.094
	Secondary	1	30 (22.1)	15 (11.0)	3 (2.2)	28 (20.6)	45 (33.1)	15 (11.0)	<0.001	0.187
		2	171 (43.1)	16 (4.0)	7 (1.8)	73 (18.4)	103 (25.9)	27 (6.8)	0.005	0.093
		3	37 (16.4)	16 (7.1)	0 (0.0)	64 (28.3)	100 (44.3)	9 (4.0)	0.038	0.094
	Vocational	1	3 (16.7)	0 (0.0)	0 (0.0)	3 (16.7)	12 (66.7)	0 (0.0)	<0.001	0.187
		2	10 (28.6)	1 (2.9)	1 (2.9)	3 (8.6)	16 (45.7)	4 (11.4)	0.005	0.093
		3	2 (8.7)	0 (0.0)	0 (0.0)	5 (21.7)	15 (65.2)	1 (4.4)	0.038	0.094
	Lower secondary	1	1 (20.0)	1 (20.0)	0 (0.0)	0 (0.0)	3 (60.0)	0 (0.0)	<0.001	0.187
		2	5 (62.5)	0	0	0	3 (37.5)	0	0.005	0.093
		3	2 (20.0)	0 (0.0)	0 (0.0)	1 (10.0)	7 (70.0)	0 (0.0)	0.038	0.094
	Primary	1	0 (0.0)	0 (0.0)	0 (0.0)	0 (0.0)	0 (0.0)	1 (100.0)	<0.001	0.187
		2	4 (40.0)	3 (30.0)	0	1 (10.0)	2 (20.0)	0	0.005	0.093
		3	4 (44.4)	1 (11.1)	1 (11.1)	2 (22.2)	1 (11.1)	0 (0.0)	0.038	0.094
Marital status	Married	1	98 (46.2)	21 (9.9)	3 (1.4)	28 (13.2)	51 (24.1)	11 (5.2)	0.012	0.157
		2	229 (49.7)	30 (6.5)	10 (2.2)	77 (16.7)	89 (19.3)	26 (5.6)	0.098	0.084
		3	75 (14.0)	32 (6.0)	4 (0.7)	151 (28.1)	255 (47.5)	20 (3.7)	0.247	0.083
	Domestic partnership/informal relationship	1	42 (28.6)	17 (11.6)	6 (4.1)	34 (23.1)	38 (25.9)	10 (6.8)	0.012	0.157
		2	148 (38.1)	19 (4.9)	8 (2.1)	80 (20.6)	102 (26.3)	31 (8.0)	0.098	0.084
		3	31 (15.1)	8 (3.9)	2 (1.0)	57 (27.7)	93 (45.2)	15 (7.3)	0.247	0.083
	Single	1	31 (29.8)	14 (13.5)	3 (2.9)	19 (18.3)	24 (23.1)	13 (12.5)	0.012	0.157
		2	132 (45.8)	17 (5.9)	4 (1.4)	80 (20.6)	102 (26.3)	20 (6.9)	0.098	0.084
		3	28 (16.2)	8 (4.6)	3 (1.7)	61 (35.3)	67 (38.7)	6 (3.5)	0.247	0.083
Health professional	Yes	1	64 (59.3)	12 (11.1)	2 (1.9)	13 (12.0)	9 (8.3)	8 (7.4)	<0.001	0.281
		2	26 (31.7)	6 (7.3)	2 (2.4)	25 (30.5)	16 (19.5)	7 (8.5)	0.041	0.101
		3	8 (9.2)	3 (3.5)	1 (1.2)	20 (23.0)	53 (60.9)	2 (2.3)	0.068	0.105
	No	1	107 (30.1)	40 (11.3)	10 (2.8)	68 (19.2)	104 (29.3)	26 (7.3)	<0.001	0.281
		2	483 (45.8)	60 (6.7)	20 (1.9)	184 (17.4)	238 (22.6)	70 (6.6)	0.041	0.101
		3	126 (15.2)	45 (5.4)	8 (1.0)	249 (30.0)	362 (43.7)	39 (4.7)	0.068	0.105
Has COVID-19 been diagnosed?	No	1	147 (37.8)	41 (10.5)	11 (2.8)	66 (17.0)	96 (24.7)	28 (7.2)	0.756	0.085
		2	423 (45.1)	53 (5.7)	20 (2.1)	159 (17.0)	218 (23.2)	65 (6.3)	0.085	0.085
		3	97 (14.2)	30 (4.4)	8 (1.2)	200 (29.2)	319 (46.6)	31 (4.5)	0.571	0.069
	Yes, in the past	1	22 (31.9)	11 (15.9)	1 (1.5)	15 (21.7)	15 (21.7)	5 (7.3)	0.756	0.085
		2	73 (40.8)	11 (6.2)	2 (1.1)	48 (26.8)	33 (18.4)	12 (6.7)	0.085	0.085
		3	37 (16.1)	18 (7.8)	1 (0.4)	68 (29.6)	96 (41.7)	10 (4.4)	0.571	0.069
	Yes, currently	1	2 (40.0)	0 (0.0)	0 (0.0)	0 (0.0)	2 (40.0)	1 (20.0)	0.756	0.085
		2	13 (65.0)	2 (10.0)	0 (0.0)	2 (10.0)	3 (15.0)	0 (0.0)	0.085	0.085
		3	0 (0.0)	0 (0.0)	0 (0.0)	1 (100.0)	0 (0.0)	0 (0.0)	0.571	0.069
Previous vaccinations	None	1	1 (4.2)	2 (8.3)	0 (0.0)	3 (12.5)	16 (66.7)	2 (8.3)	<0.001	0.358
		2	13 (23.2)	3 (5.4)	0 (0.0)	6 (10.7)	33 (58.9)	1 (1.8)	<0.001	0.255
		3	4 (6.6)	1 (1.6)	0 (0.0)	7 (11.5)	49 (80.3)	0 (0.0)	<0.001	0.157
	Only mandatory	1	58 (23.1)	21 (8.4)	6 (2.4)	59 (23.5)	86 (34.3)	21 (8.4)	<0.001	0.358
		2	250 (35.7)	38 (5.4)	12 (1.7)	155 (22.1)	190 (27.1)	55 (7.9)	<0.001	0.255
		3	79 (12.9)	35 (5.7)	6 (1.0)	185 (30.1)	280 (45.6)	29 (4.7)	<0.001	0.157
	Mandatory and recommended	1	112 (59.6)	29 (15.4)	6 (3.2)	19 (10.1)	11 (5.9)	11 (5.9)	<0.001	0.358
		2	246 (64.6)	25 (6.6)	10 (2.6)	48 (12.6)	31 (8.1)	21 (5.5)	<0.001	0.255
		3	51 (21.2)	12 (5.0)	3 (1.2)	77 (32.0)	86 (35.7)	12 (5.0)	<0.001	0.157

## Data Availability

The data presented in this study are available on request from the corresponding author.

## Supplementary Materials

The following are available online at https://www.mdpi.com/article/10.3390/vaccines10030381/s1, Table S1: The analysis of covariance (ANCOVA) of potential confounding factors.
