# Supplementary material for: Assessment of Attitudes, Main Concerns and Sources of Knowledge Regarding COVID-19 Vaccination in Poland in the Unvaccinated Individuals—A Nationwide Survey
Source: Vaccines (Basel). 2022 Mar 2;10(3):381. doi: 10.3390/vaccines10030381 (PMC8955026; doi:10.3390/vaccines10030381)
Supplement: Supplementary file 1 [file vaccines-10-00381-s001.zip › vaccines-1578531-supplementary.pdf]

Supplementary Materials

Table S1: The analysis of covariance (ANCOVA) of potential confounding factors

Concern	Confounding factor	F	p
**Vaccine adverse events**	Sex	0.270	0.764
	Age	0.918	0.702
	Place of residence	1.800	0.095
	Level of education	0.453	0.889
	Marital status	**3.410**	**0.009**
	Health professional	**3.243**	**0.039**
	Previous vaccinations	**3.764**	**0.005**
**Microscopic**	Sex	2.330	0.098
	Age	1.037	0.382
	Place of residence	1.850	0.085
	Level of education	0.615	0.766
	Marital status	**3.450**	**0.008**
	Health professional	**3.385**	**0.034**
	Previous vaccinations	**4.013**	**0.003**
**Transport**	Sex	0.050	0.956
	Age	0.939	0.651
	Place of residence	1.410	0.209
	Level of education	1.435	0.177
	Marital status	2.290	0.057
	Health professional	**4.952**	**0.007**
	Previous vaccinations	1.124	0.343
**Vaccine efficacy**	Sex	2.440	0.087
	Age	0.861	0.832
	Place of residence	1.360	0.228
	Level of education	1.059	0.389
	Marital status	**4.010**	**0.003**
	Health professional	0.264	0.768
	Previous vaccinations	1.950	0.100
**Conspiracy**	Sex	0.020	0.984
	Age	1.008	0.461
	Place of residence	1.460	0.188
	Level of education	1.170	0.314
	Marital status	0.310	0.870
	Health professional	**3.950**	**0.019**
	Previous vaccinations	**6.260**	**<0.001**
**Other**	Sex	0.140	0.869
	Age	0.682	0.993
	Place of residence	1.230	0.287
	Level of education	0.626	0.757
	Marital status	0.380	0.822
	Health professional	1.520	0.218
	Previous vaccinations	1.040	0.387
